# Teaching and placement of dental amalgam restorations at South African dental schools

**DOI:** 10.3389/froh.2023.1118361

**Published:** 2023-07-19

**Authors:** Ameera Y. Essa, Suwayda Ahmed, Achmat Dyason, Farzana Karjiker, Razia Z. Adam

**Affiliations:** Department of Prosthodontics, Faculty of Dentistry, University of the Western Cape, Cape Town, South Africa

**Keywords:** dental amalgam, phase-out, direct posterior restorations, dental schools, clinical teaching

## Abstract

**Introduction:**

This paper set out to investigate the relationship between teaching and clinical practice of direct posterior restoration placement at tertiary dental institutions in South Africa.

**Methods:**

A cross-sectional study using a mixed methods approach was conducted, and all the South African dental schools were invited to participate. The quantitative phase of the study analyzed the Conservative Dentistry department's records of direct restorations at a single dental school. The number of amalgam and tooth-colored restorations placed by students in the fourth and fifth year of the program from 2004 to 2019 were compared. During the qualitative phase, semi-structured interviews were held with staff from the four dental schools involved with the teaching of direct posterior restorations.

**Results:**

The predominant direct posterior restoration placed in student clinics over a 15-year period at a South African dental school was tooth colored (75%). Teaching times do not correspond to a decrease in the placement of dental amalgam restorations for both one-surface and two-surface posterior restorations and a concomitant increase in the tooth-colored restorations.

**Discussion:**

Academic staff involved in teaching identified that South Africa's ratification of the Minamata Convention has consequences for dental education and training. However, all schools reported that dental amalgam would continue to be taught in the absence of an appropriate alternative.

## 1. Introduction

In 2019, South Africa ratified the Minamata Convention. The objective of the Convention is to “protect human health and the environment from anthropogenic emissions and releases of mercury and mercury compound measures” (1). The United Nations Environmental Program estimated that dental amalgam contributed between 21%–32% and 9%–13% of air and water emissions in Europe (2). Mercury emissions result from dental amalgam disposal, waste, removal, and cremation. The Minamata treaty necessitates the phase-down of mercury-added products used in research, health, and dental amalgam. The phase-down of amalgam impacts restorative dentistry considerably since appropriate amalgam alternatives are not available.

The presence of mercury in blood, urine, and tissues have been positively correlated to the number of dental amalgam restoration surfaces (3). The US Food and Drug Administration (USFDA) evaluated “34 peer-reviewed, primary research studies selected for their scientific merit and their potential to provide the most significant and new information regarding health risks associated with exposure to mercury vapor”. Two recent clinical trials and other retrospective studies did not support claims that mercury exposure via dental amalgams led to adverse biological outcomes associated with neuropsychological function, low birth weight, multiple sclerosis and Alzheimer's disease (4, 5). The report concluded that dental amalgam did not pose a health threat.

Global bodies such as the World Health Organization (WHO), World Dental Federation (FDI), International Association for Dental Research (IADR), and UK organizations like the British Dental Association (BDA) have argued for a gradual decrease in the use of dental amalgam rather than a complete ban (1).

It is imperative to understand the impact of a phase-down of dental amalgam through all stakeholders. This includes the teaching practices at dental schools and the attitude of dental academics. With the improvement in the properties of composite resins and dentine bonding agents, the use of composite resin in posterior restorations has increased (6–9). The shift in preference of composite resin over amalgam for posterior restorations is influenced by an increase in the practice of minimally invasive restorative techniques, an increased demand for aesthetic restorations, and patients' concern with mercury toxicity, despite the lack of evidence to support this claim (6, 7, 10).

The curriculum for operative dentistry focused on amalgam for posterior restorations and composites for anterior restorations, but in the mid-1990 s the choice of material for posterior restorations shifted from amalgam to composite resin and this trend has continued to the present day (8). Clinical teachers are tasked with examining current evidence, so that professional education is imparted to the undergraduate students, which is consistent with science (evidence based), and also meeting patients' needs (7).

The dental curriculum is influenced by factors such as scientific research, socio-economic and political policies, as well as public opinion. Dental faculties revise curricula to ensure that innovative teaching techniques and technologies are embraced, and that graduates gain appropriate knowledge and skills to enable safe and effective practice (11). Teaching trends in the United Kingdom (UK) and North America are geared toward the teaching and placement of posterior composite restorations, and some dental schools dedicate a small percentage of time teaching amalgam placement (10).

A study by Ben-Gal et al. (6) analyzed all restorations placed by undergraduate students to determine if there was an increase in the placement of posterior composite restorations. Their results showed that there was a marked increase in the placement of posterior composites. The researchers also found that preference shared indicated that the younger clinical teachers favored posterior composites, while the older clinical teachers leaned toward posterior amalgam restorations. In a 2006 survey of dental schools in the United States (US), Ireland and UK, 30% of posterior restorations placed were composite resin restorations. In a survey of Canadian dental schools, 50% of posterior restorations were composite resin (12).

Otenga and Mjör (8) investigated the teaching volume (lectures, preclinical sessions, etc.) and the number of restorations placed by undergraduate students. Their results showed that in 2005, most of the didactic and lab teaching focused on amalgam, and accordingly, amalgams were placed. One year later, in 2006, when students reached the clinical component of the undergraduate course, an increase in posterior composites was noted. The conclusions were that a closer relationship should exist between what is taught and what is exercised by students.

A recent paper explored dental amalgam teaching in dental therapists' training at a public university in South Africa and reported that dental amalgam is still integral to dental education (13). There is no current data regarding dental amalgam teaching practices for dentists in South Africa. The position of the Committee of Dental Deans in South Africa (March 2015) emphasized the need to continue to teach both amalgam and composite techniques, and students should be taught placement of composites using correct techniques. The purpose of this research is to investigate the relationship between teaching and direct posterior restoration placement at tertiary dental institutions in South Africa.

## 2. Materials and methods

The cross-sectional study used a mixed methods approach with two phases, namely: a quantitative analysis of student records of the number of dental amalgam and tooth-colored restorations placed over a 15-year period at a South African dental school; and a qualitative analysis of interviews with dental academics at South African universities responsible for the teaching of direct posterior restorations. The research was approved by the Biomedical Research and Ethics Committee of the University of the Western Cape (BM20/10/8).

### 2.1. Phase one: quantitative study

This was a retrospective record-based study. In the Department of Conservative Dentistry at the conveniently selected dental school, students are required to have a recommended number of clinical procedures.Each procedure has a specific code as specified in the Government Gazette (14) (see Table 1) These records are collected by the department in an Excel programme custom-made for the purpose. A cross tabulation allows selection of a specific year group and specific procedure. The clinical student records for the direct posterior restorations of the fourth year Bachelor of Dental Surgery (BDSIV) dental students and the fifth year Bachelor of Dental Surgery (BDSV) dental students from the Department of Conservative Dentistry were analyzed for the period 2004–2019. Departmental records were also used to collate preclinical and clinical requirements and educational philosophy with regards to the use of amalgam and tooth-colored restorations in posterior teeth. Frequency distributions for each code as seen in Table 2 was calculated.

**Table 1:** Codes for posterior direct restorations.

8,341	One surface amalgam restoration
8,342	Two surface amalgam restoration
8,343	Three surface amalgam restoration
8,367	One surface tooth coloured posterior restoration
8,368	Two surface tooth coloured posterior restoration
8,369	Three surface amalgam posterior restoration

**Table 2:** Number of posterior direct restorations.

Year	Amalgam restorations (posterior) (*n*)	Percentage of total restorations (%)	Tooth coloured restorations (*n*)	Percentage of total restorations (%)	Total number of restorations (*n*)
2004	408	42,9	542	57,1	950
2005	1,658	32,3	3,482	67,7	5,140
2006	877	36,9	1,498	63,1	2,375
2007	1,469	31,7	3,162	68,3	4,631
2008	1,518	37,0	2,586	63,0	4,104
2009	1,284	28,5	3,226	71,5	4,510
2010	1,787	34,8	3,353	65,2	5,140
2011	1,160	27,2	3,106	72,8	4,266
2012	825	22,2	2,894	77,8	3,719
2013	532	16,7	2,649	83,3	3,181
2014	581	16,6	2,924	83,4	3,505
2015	364	15,8	1,933	84,2	2,297
2016	352	14,6	2,061	85,4	2,413
2017	570	13,6	3,606	86,4	4,176
2018	244	8,6	2,605	91,4	2,849
2019	118	5,5	2,021	94,5	2,139
Total	13,765		43,410		55,395

### 2.2. Phase two: qualitative study

Purposefully selected heads of departments at the four dental schools in South Africa were contacted via email to introduce the study to them, and to request staff participation in the study. The heads of departments then disseminated the emails to the responsible module coordinators. Staff responsible for teaching the content in the undergraduate operative or restorative programs who expressed willingness to participate in the study, were invited to join in semi-structured interviews to elicit an overview of teaching times, preclinical and clinical requirements, and educational philosophy with regards to the use of amalgam and composite resin restorations in posterior teeth.

Eight (8) participants expressed their willingness to participate in the study and were invited to attend using the Google Meet forum. All participants completed informed consent prior to the interviews via email. An interview guide was compiled informed by the analysis of the quantitative data as well current literature.. Interview data was transcribed using the Otter programme and analysed using the thematic analysis approach (15). Participant validation, peer debriefing and reflexivity was used to triangulate data and ensure validity and reliability.

## 3. Results

A total of 55,395 direct posterior restorations were placed during 2004–2019 in student dental clinics at a South African dental school. Of these, about twenty-five percent (*n *= 13,747) were dental amalgam restorations and seventy-five percent (*n *= 41,648) were tooth-colored restorations.

**Figure 1:**
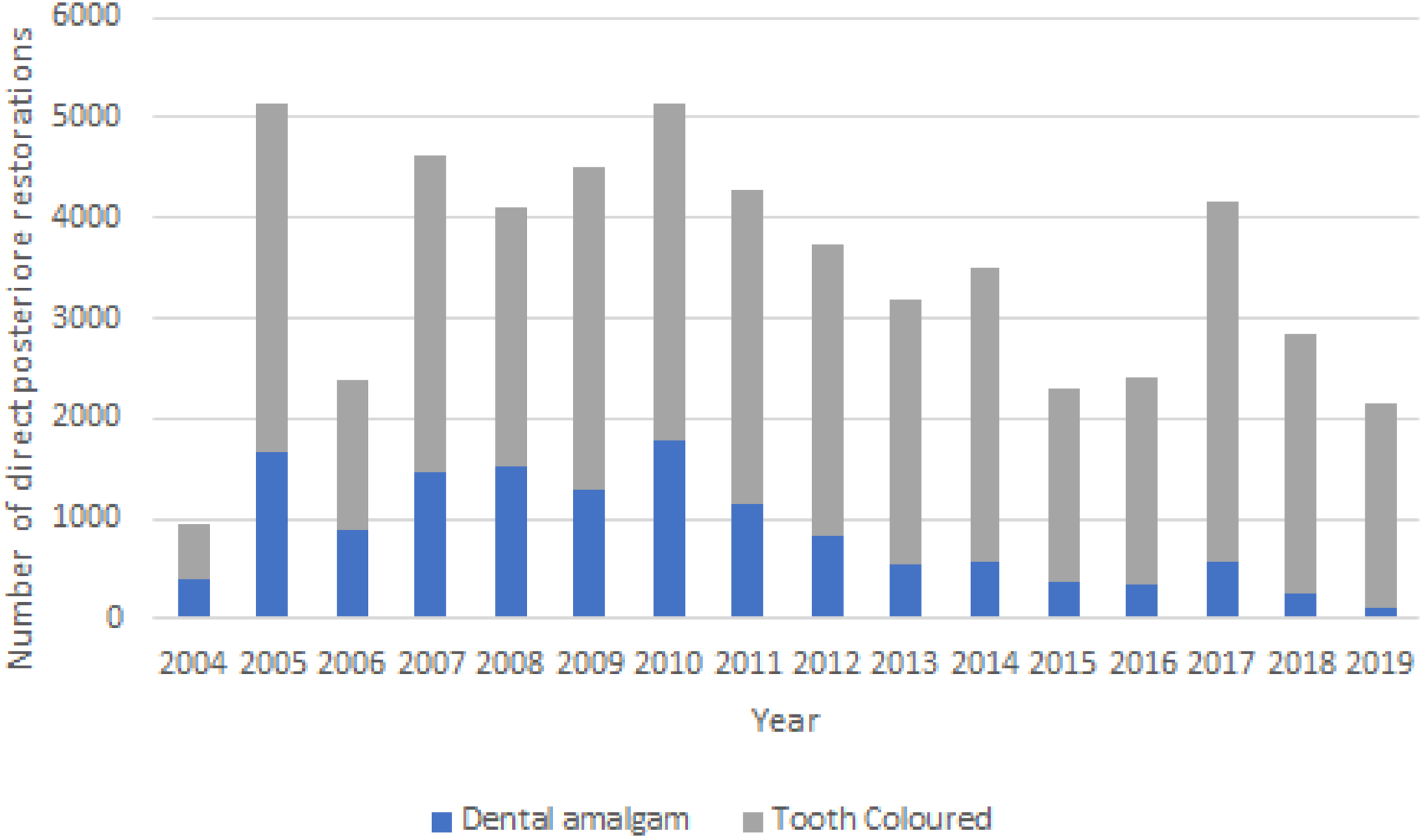
Number of direct posterior restorations placed in a dental student clinic (2004–2019).

Analysis of the data revealed that from 2004 the use of dental amalgam as a direct posterior restorative material dropped dramatically from Forty-two point nine percent to five point five percent in 2019 as seen in Figure 1. Tooth-colored direct restorations, which can be assumed consisted predominantly of resin composite restorations, accounted for fifty-seven percent of all direct posterior restorations in 2004 and increased significantly toninety-five percent in 2019.

**Figure 2:**
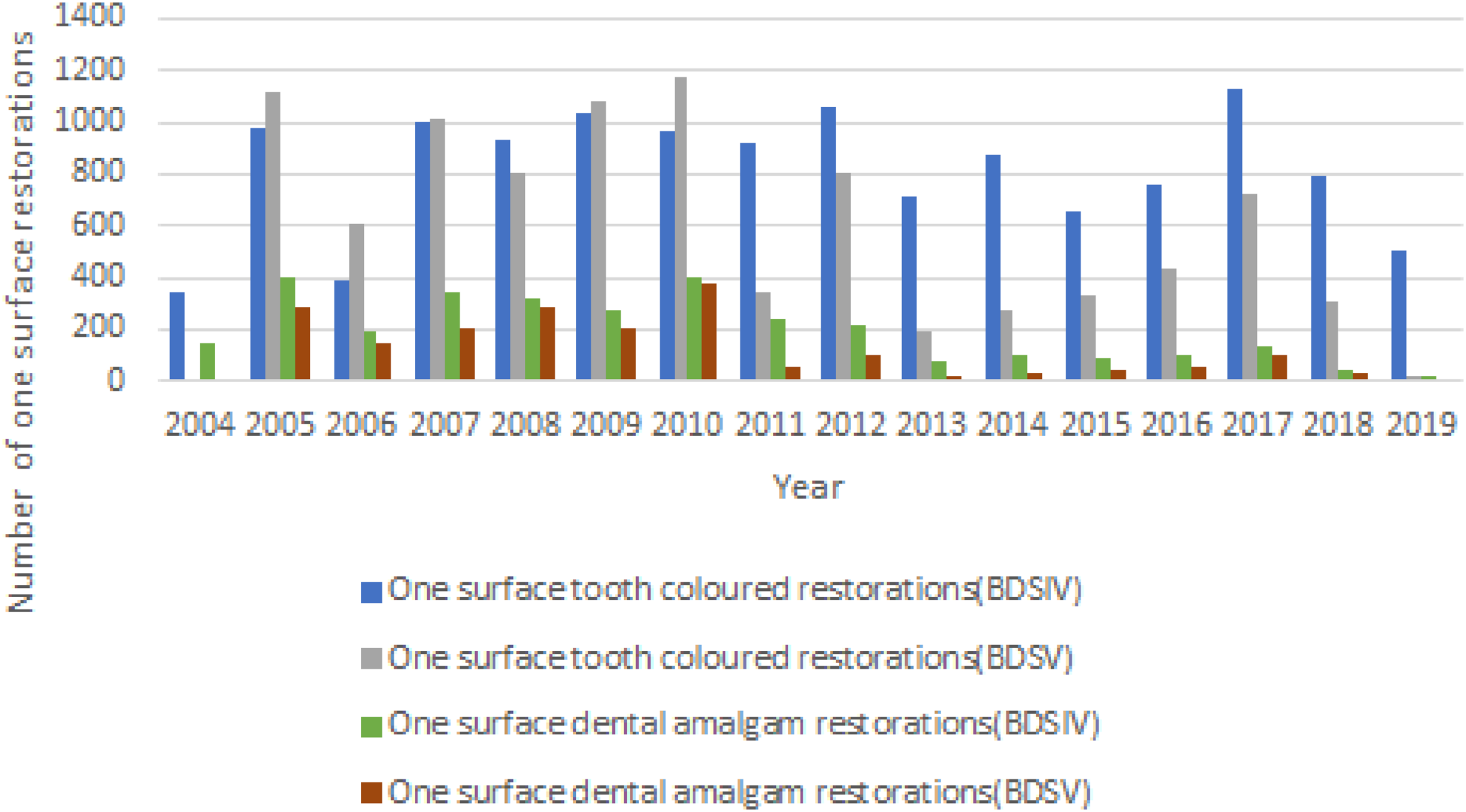
One surface restorations placed by fourth and fifth year dental students during 2004–2019.

As illustrated in Figure 2, we compared the number of one-surface dental amalgam restorations (8,341) with the one-surface tooth-colored restorations (8,367) placed by students enrolled in the fourth year Bachelor of Dental Surgery (BDSIV) and the fifth year Bachelor of Dental Surgery (BDSV) dental Programmes.

**Figure 3:**
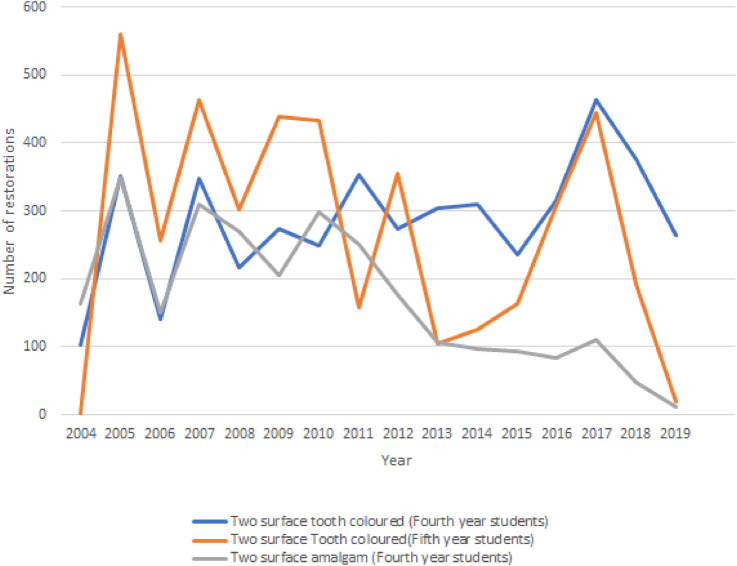
Two surface restorations placed by fourth and fifth year dental students (2004–2019).

The majority of tooth-colored restorations (99%) and dental amalgam restorations (97%) were placed in the fourth year of the program. These findings are similar for two surface posterior restorations placed by the two different year groups as seen in Figure 3.

From 2010 onwards, a downward trend in the placement of one-surface dental amalgam restorations is evident.

### 3.1. Results from the interviews

Invitations to participate were sent to all four dental schools via email and via the Google Meet Forum, and followed up every two weeks for two months. All dental schools training dentists participated in the study except for one which did not respond to any of the invites.

#### 3.1.1. Impact of minamata convention on dental schools

All respondents were familiar with the Minamata Convention and confirmed there were implications for dental training. A phase-out approach was adopted at all dental schools except for one.

“*We've already started implementing changes to accommodate [the Minamata Convention]* … *preclinical teaching. What we've done with our students is, we have in actual fact decreased the amount of amalgam procedures that they themselves have to practice”* (Respondent 2).

“…*our teaching time now, we did not reduce it since that time, because there was an agreement. It was about five years ago that all universities came together and said, we will still continue doing the teaching and training of amalgam because it's important for the hand skills, especially preclinical because you know, composite cavities, designed by caries but amalgam there is specific designs that a student needs to do, so we haven't reduced it per se”* (Respondent 3).

Respondent 2 also expressed a concern that students may have a skills deficit with regards to clinically handling dental amalgam if it is no longer taught. The respondent raised the importance of ensuring that topics such as removal of dental amalgams and repairing of dental amalgams be introduced.

#### 3.1.2. Teaching of direct posterior restorations

Less time was spent on teaching dental amalgam preclinically as there were fewer types of restorations that needed to be taught (class I, class II, and class V) and the recommended number of clinical procedures were reduced. Consequently, the teaching time of the placement of posterior composite resin restorations was increased.

“*So definitely, much more time spent on composites and your bonding systems itself on; so it's much more time, it's very difficult to give a number, but I would say at least 80%–90% on composite, so 10% or more”* (Respondent 3).

“…*the preclinical undergraduate training. Yeah. I think it's only 25% [for dental amalgams]. I have increased the number of composites that they have to do and decrease[d] the number of amalgams”* (Respondent 2).

“*We've changed our preclinical requirements, not removing amalgam by just maybe replacing some of the amalgam exercises with composite instead. Okay. So, an example of that would be one of the two surface restorations that they would do. Instead of doing three amalgams and two composites, they now do two amalgams and three composites”* (Respondent 1).

#### 3.1.3. Dental amalgam and relevance in the South African context

Respondents differed in opinion on the relevance of dental amalgam in South Africa. One respondent felt that whilst very few general dentists used dental amalgam in their private practices, there was a need to teach the skill as dentists in public health did not have access to the resources needed to place composite restorations. Another respondent commented that dental amalgams as used in complex restorations were an affordable option for patients who required indirect restorations but could not afford them.

“*In real private practice or in public clinics, amalgam is still material that's used on a daily basis. We know in the real world you still need amalgam”* (Respondent 3).

“*In the public sector, it was still more feasible to use amalgam in terms of cost and longevity, you know, in patients. And so that's why it's retained its place in our curriculum”* (Respondent 1).

“….*to be equipped with everything necessary to do quality, tooth-colored materials from having rubber dam[s] available to proper curing lights to the instruments, burs that are required. I think that's probably one of the bigger challenges”* (Respondent 1).

“*But I do reserve judgment in terms of a complete phase out, especially in our economic climate, with respect to patients and affordability*” (Respondent 2).

## 4. Discussion

In the context of South Africa ratifying the Minamata Convention in 2019, current data was needed to contextualize the teaching and the placement of dental amalgam in South African dental schools. While dental amalgam is a durable restorative material, evidence on its environmental impact due to the material's use has led to the calls for a phase-down of dental amalgam use (16, 17). Interestingly though, a recent paper has reported that resin-based composites have the highest global warming potential (16).

A clear increase in the placement of posterior tooth-colored restorations was evident can be attributed to a variety of factors including ease of placement, patient preference, aesthetics, and the trend towards minimally invasive dentistry (8, 10, 11, 13). This large increase in tooth colored posterior restorations correlates with data from the UK and North America which has also shown an increase over a 10-year period (12).

The majority of one-surface posterior direct restorations were placed by fourth year Bachelor of Dental Surgery (BDSIV) dental students between 2004 and 2019 (Figure 2). This could likely be attributed to the fifth year Bachelor of Dental Surgery (BDSV) dental students concentrating on the clinical requirements for indirect restorations.

The results of the qualitative part of the study, which consisted of three respondents, showed that the majority of dental schools in South Africa are already implementing the recommendations of the Minamata Convention's phase-down in the use of dental amalgams. While universities have decreased the quota/number of amalgam restorations required to be placed by dental students, two respondents raised the concern that placement of amalgam restorations is necessary for the development of certain “hand-skills” in dentistry, especially in the pre-clinical environment. The concern that a complete shift to tooth-colored restorations would negatively impact the students' skills development was raised by a recent South African study (13). The skills development for dental amalgam restorations is due to the need for a specific cavity design whereas cavity design for tooth-colored restorations follows a caries-directed approach.

In 2015, the Committee of Dental Deans resolved to continue to teach the placement of dental amalgam restorations without the availability of an alternative material. This document was used as a motivation for not adjusting the recommended clinical requirements for dental amalgam at one of the schools.

While variations in the global recommendations exist, the current practices at South African dental schools are in line with teaching practices in Canadian training facilities which varied between less than twenty-five percent and between twenty-five and fifty percent of teaching time being dedicated to amalgam restorations (7). A decrease in the number of pre-clinical requirements for dental amalgams was also noted with tooth-colored restorations having replaced some of the previous amalgam quotas in South African dental schools. Similar findings were reported by an Australian study conducted in 2018, where a consensus was reached that dental amalgam should still be taught but the focus must shift to adhesive restorations (18). There is not much uniformity amongst the universities regarding the exact teaching practices of amalgam posterior restorations, however most of the universities are moving towards a scale-down of amalgam quotas.

In South Africa, the socio-economic status of many patients presenting to the public sector for restorations must be considered. It is important to highlight the increased capital cost associated with tooth-colored restorations such as curing lights, bonding systems, rubber dams and burs, as well as increased maintenance costs (1, 10). Dental amalgam remains an affordable option for many patients requiring complex restorative treatments, including those requiring indirect restorations without the adequate funds. Thus, the complete replacement of amalgam by composite is not recommended, as correlated by other studies (11, 13).

In terms of longevity and cost of restorations, amalgam has been noted to display superior performance over tooth-colored restorations. In cases where composite resin is contraindicated, dental amalgam may be an alternative (7). While few general dentists in South Africa still place dental amalgam restorations in private clinics, it remains important for the required skills to be taught at university, especially as those in public service require the necessary training.

Emphasis on teaching of repair and refurbishment was also raised by the Australian study (17).The necessity for educating students on the repair and removal of amalgam was also mentioned. This must be addressed at all universities in South Africa as it is in keeping with the practice of minimally invasive dentistry (10, 19).

A limitation of this study is that the quantitative data were obtained from one university only and cannot necessarily be indicative of the practices at all the other dental schools in South Africa.

## 5. Conclusion

While the pre-clinical and clinical teaching requirements of amalgam restorations has been decreased at all universities that participated in this study, the economic climate in South Africa is not conducive to a complete phase-out of amalgam restorations. Thus, the complete phase-out of amalgam restorations is unanimously discouraged due to affordability concerns for the masses. Furthermore, universities have the responsibility to ensure that undergraduate students are well-informed about the placement and repair of amalgam restorations to conserve tooth structure and decrease the environmental concerns surrounding amalgam disposal.

## Data Availability

The original contributions presented in the study are included in the article/supplementary materials, further inquiries can be directed to the corresponding author.
